# Markers of Anemia in Children with Type 1 Diabetes

**DOI:** 10.1155/2018/5184354

**Published:** 2018-05-31

**Authors:** Ewa Rusak, Anna Rotarska-Mizera, Piotr Adamczyk, Bogdan Mazur, Joanna Polanska, Agata Chobot

**Affiliations:** ^1^Department of Pediatrics and Children's Diabetology, Medical University of Silesia, 40-752 Katowice, Poland; ^2^Data Mining Division, Faculty of Automatic Control, Electronics, and Computer Science, Silesian University of Technology, 44-100 Gliwice, Poland; ^3^Department of Pediatrics, School of Medicine with the Division of Dentistry in Zabrze, Medical University of Silesia, 40-752 Katowice, Poland; ^4^Department of Pediatric Gastroenterology and Hepatology, Clinical Hospital No. 1, 41-800 Zabrze, Poland

## Abstract

**Aim:**

The aim of the study was to assess markers of anemia in type 1 diabetes (T1D) children, compare them to results obtained in the control group, and estimate their relation to BMI SDS.

**Methods:**

94 (59% ♀) T1D children without other autoimmune disorders, aged 12.5 ± 4.1 years, T1D duration: 4.2 ± 3.6 years, HbA1c 7.3 ± 1.5% (57 ± 12.6 mmol/mol). Sex- and age-matched controls (43 children). In all children, anthropometric measurements, the blood count, iron turnover parameters, and vitamin B12 concentration were taken.

**Results:**

T1DM children had significantly higher red cell distribution width (RDW) (13.6 versus 12.6%; *p* < 0.001), hepcidin (0.25 versus 0.12 ng/ml; *p* < 0.001), and vitamin B12 concentrations (459 versus 397 pg/ml; *p* < 0.01) and lower TIBC (59.09 versus 68.15 *μ*mol/l; *p* < 0.001) than in the control group. Logistic regression revealed that RDW, TIBC (both *p* < 0.001), and hepcidin (*p* < 0.05) significantly differentiated both groups. In T1DM children, BMI SDS negatively correlated with vitamin B12 (*p* < 0.01) concentration and mean corpuscular hemoglobin concentration (*p* < 0.05) and positively with TIBC (*p* < 0.01) and HbA1c (*p* < 0.001).

**Conclusions:**

Patients and controls differed especially in terms of RDW and TIBC. In studied T1DM children, BMI SDS was associated to iron metabolism parameters and vitamin B12 concentration.

## 1. Introduction

Type 1 diabetes (T1D), because of the rising incidence, remains a challenge for diabetologists as well as for pediatricians and general practitioners, who more and more often face the challenge to take optimal care for young patients with this disease [1, 2]. With time, systemic consequences such as anemia may develop in children with T1D. In order to diagnose this condition, it is enough to perform a simple blood count and to search for its causes; besides the standard assessment of the iron serum concentration, it is worth to broaden the diagnostics by the assessment of the parameters of iron metabolism. The interpretation of such results is not easy. Anemia in T1D may have a complex, multifactorial background [3]. Among the most common causes of anemia in the course of T1D in children is iron deficiency, which will present as anemia with microcytosis in the blood count. Its prevalence is higher among T1D patients in comparison to people without diabetes [4]. However, anemia that accompanies inflammation may also present with microcytosis [5]. On the other hand, in cases of diabetic nephropathy, because of insufficient erythropoietin production, normocytic anemia develops [6]. If diagnostic procedures rule out the most common causes, cooccurrence of other autoimmune diseases (thyroiditis, celiac disease, Addison's disease, and autoimmune atrophic gastritis) that may be accompanied by anemia of various morphologies should be taken into account [3]. The variability of causes and their mutual overlapping significantly impedes the interpretation of the obtained results, but the proper diagnosis conditions optimal treatment and allows avoiding systemic complications. Additionally, adequate interpretation of the HbA1c measurement, that is routinely performed during diabetes control visits, requires also to know the patient's serum iron concentration, because the presence of iron deficiency anemia correlates with higher HbA1c values [7]. It is interesting that even though the value of a performed blood count is well known as well as the multiplicity of information it gives, there are no recommendations whether and when it should be carried out in T1D patients—neither in the guidelines published by Diabetes Poland (2018) nor those of the International Society for Pediatric and Adolescent Diabetes [8, 9]. Until present, there have been a few publications assessing the blood count, iron metabolism parameters, and vitamin B12 levels in children with T1D, which most often use other T1D individuals as a control group. To our knowledge comparisons to healthy peers are lacking.

According to our research hypothesis, children with T1D differ from healthy peers in terms of blood count, iron metabolism parameters, and vitamin B12 levels. Therefore, the aim of the study was to assess the abovementioned parameters in T1D pediatric patients with no other autoimmune diseases and compare them to results obtained in a group of healthy children as well as estimate their relation to chosen factors, among others: anthropometric measurements, sex, glycemic control, and disease duration. Confirming the research hypothesis would be a strong premise to monitor red blood cell parameters and—depending on the results—adequate broadening of the diagnostic tests.

## 2. Material and Methods

### 2.1. Patients

The study group included 94 children with T1D (55 females) (Diabetic Outpatient Clinic, Upper Silesian Centre for Child's Health in Katowice, Poland). The blood samples were collected during the routine control visit in the Outpatient Clinic. Their mean age at study time was 12.5 ± 4.1 years (SD) (ranging from 3 to 19 years). Mean duration of T1D was 4.2 ± 3.6 years (SD) (0.08–15.7 years). For the purposes of statistical analysis, the percent of life with diabetes was calculated (ratio of disease duration to age, expressed in %). The mean glycated hemoglobin A1c (HbA1c) at the day of the study is equaled to 7.3 ± 1.5% (57 ± 12.6 mmol/mol).

In all patients, physical examination with auxological assessment was performed. Routine laboratory tests (screening for autoimmune concomitant diseases and complications) were performed according to recent Diabetes Poland and ISPAD guidelines [8, 9].

Patients with clinical and/or laboratory signs of other than T1D autoimmune diseases (celiac disease, thyroiditis) or infection were excluded from the study. Furthermore, children enrolled for this study, according to their medical records, did not have chronic diabetes complications.

### 2.2. Controls

The control group comprised 43 children (24 females, 19 males) that were age- and sex-matched to the individuals from the study group. They were recruited from patients admitted to the Clinical Hospital No. 1 in Zabrze for nocturnal enuresis or arterial hypertension diagnostics in whom hospital observation and diagnostic procedures allowed to exclude any organic disease that could influence the parameters assessed in this project. These children had no infection, autoimmune diseases, or family history of such diseases.

### 2.3. Anthropometric Assessment

Anthropometric measurements carried out in all children included weight (kg) and height (cm). Using these values, the body mass index (BMI) was calculated using the standard equation (the body mass in kilograms divided by the square of the body height in meters). To allow comparisons, weight, height, and BMI were expressed as standard deviation scores (SDS).

### 2.4. Laboratory Measurements

Patients and controls had blood samples taken to perform the blood count (1.6 ml) and carry out biochemical measurements (5 ml)—iron, ferritin, transferrin, hepcidin, vitamin B12 concentrations, and total iron-binding capacity (TIBC). Additionally, children with T1D had their HbA1c and fetal hemoglobin (HbF) measured (capillary blood 10 *μ*g). The blood samples were taken between 7 and 9 am while the children were on fasting. Peripheral blood count (RBC: red blood cells (10 > 6/*μ*l), MCV: mean corpuscular volume (fl), MCHC: mean corpuscular hemoglobin concentration (g/dl), HGB: hemoglobin (g/dl), HCT: hematocrit (%), RDW: red cell distribution width (%) measured using Sysmex XT-2000i (Kobe, Japan)) and biochemical parameters (by means of immunochemical methods using commercial sets (Roche Diagnostics GmbH, Mannheim, Germany) and the cobas 6000 device (Hitachi, Japan)) were measured in the Central Laboratory of the Clinical hospital No. 1 in Zabrze, Poland. Normal ranges, lower limits of detection (LLD), indirect precision (IP), and repeatability (R) of the measured biochemical parameters are the following: vitamin B12 >150 pg/ml (LLD 100 pg/ml, IP 4.5%, R 3.5%), transferrin 2–3.6 g/l (0.1 g/l, IP 3.0%, R 1.5%), ferritin 20–200 *μ*g/l (LLD 5 *μ*g/l, IP 2.2%, R 1.2%), iron 5.83–24.5 *μ*mol/l (LLD 0.90 *μ*mol/l, IP 1.5%, R 1.0%), and TIBC 55–75 *μ*mol/l (LLD 6 *μ*mol/l, IP 4.8%, R 1.2%). Hepcidin concentration was determined using an enzyme-linked immunosorbent assay (EIA Science Co., Wuhan, China); normal range: 0.187 to 12 ng/ml (LLD 0.00625 ng/ml, IP 9.8%, R 6.1%). HbA1c assessment was carried out using a DCCT (Diabetes Control and Complications Trial) reference method; normal range < 6% (<42 mmol/mol).

Anemia was defined as reduction in hemoglobin concentration and/or red blood cells compared to norms adopted for a specific child's age and gender.

The project was carried out between August 2014 and June 2015 as a part of the MNiSW grant number IP2012 007672. The study and its protocol obtained a positive opinion of the Ethical Committee of the Medical University of Silesia in Katowice, Poland. Parents or lawful caregivers and patients aged 16 years or more gave written consent for the abovementioned diagnostic procedures and for participation in this research project.

### 2.5. Statistical Analysis

Statistical analyses were performed using the R software (http://www.bioconductor.org/).

For all of the analyzed parameters, the following descriptive statistics were determined: number (*N*), median, upper and lower quartile (*Q*1 and *Q*3), minimal (min) and maximal (max) value, mean, and standard deviation (SD).

Tukey's criterion was used to identify outlying values. Lilliefors parametric test was used to assess the normality of the distribution. The variance homogeneity hypothesis was tested by means of *F* or Bartlett statistic. Comparative analysis of normally distributed variables was carried out by analysis of variance (ANOVA) or Student's *t*-test depending on the group number. In cases of other types of distribution, the nonparametric ANOVA Kruskal-Wallis and Mann–Whitney *U* test were employed. For post hoc comparisons, we used the Tukey-Kramer test or its nonparametric alternative. The logistic regression technique with the forward feature selection procedure was applied during the construction of the binary classifier. Akaike information criterion supported by Bayes factor principle adjudged on the final model (BF > 3.1623) [10]. Correlations between two continuous variables were assessed by means of the Pearson's or Spearman's correlation coefficients. To identify the most influential explanatory variables and estimate the adjusted model coefficients, the multivariate linear regression method combined with forward feature selection algorithm and likelihood ratio test (LRT) were used. Discrete variables were analyzed using the *χ*^2^ or likelihood ratio *G* test. Statistical significance was considered at *p* < 0.05.

## 3. Results

In patients as well as in controls, the mean values of all blood count, iron metabolism parameters, and vitamin B12 results were within normal range—mean values and their 95% confidence intervals are presented in Table 1. Despite that both studied groups differed in terms of the RDW, which was significantly higher in children with T1D (13.6 (95% Cl 13.3, 13.8) versus 12.6 (95% Cl 12.4, 12.9) *p* < 0.001) (Table 1). Analysis of biochemical parameters revealed higher hepcidin (0.25 (95% Cl 0.20, 0.29) versus 0.12 (95% Cl 0.10, 0.14) *p* < 0.001) and vitamin B12 concentrations (459 (95% Cl 419, 500) versus 397 (95% Cl 356, 437) *p* < 0.01) as well as significantly lower TIBC values (59.09 (95% Cl 57.51, 60.67) versus 68.15 (95% Cl 65.29, 71.02) *p* < 0.001) in T1D patients (Table 1). Logistic regression allowed to determine RDW, TIBC (for both *p* < 0.001), and hepcidin (*p* < 0.05) as parameters significantly differentiating the study and the control group with 89% sensitivity and 77% specificity (Tables 2 and 3).

**Figure 1:**
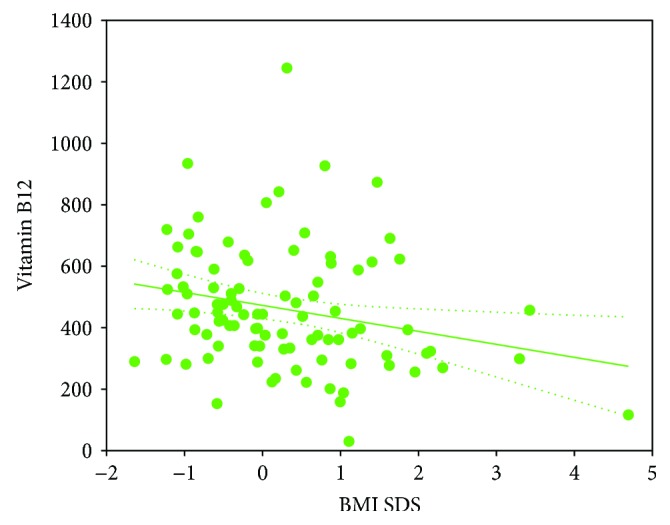
Correlation between serum vitamin B12 concentration (pg/ml) and body mass index standard deviation score (BMI SDS) in type 1 diabetes children (*r* = −0.27, *p* < 0.01).

**Figure 2:**
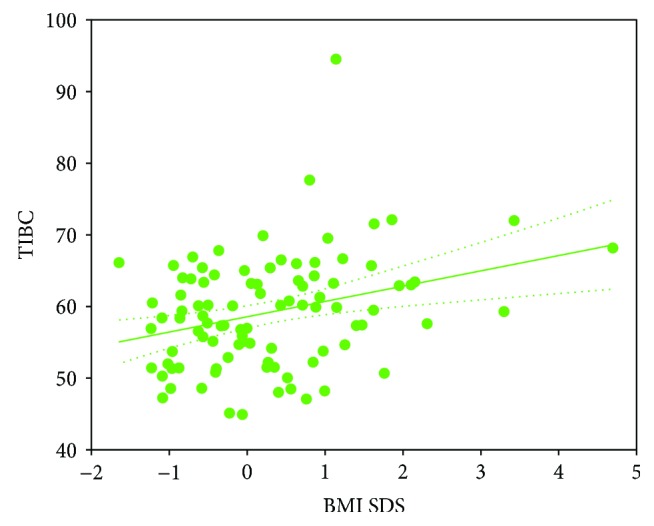
Correlation between total iron-binding capacity (TIBC) (*μ*mol/l) and body mass index standard deviation score (BMI SDS) in type 1 diabetes children (*r* = 0.28, *p* < 0.01).

Among patients with T1D, hepcidin correlated positively with RDW (*r* = 0.27, *p* < 0.01). There was also a negative relation between RDW and HGB (*r* = −0.24, *p* < 0.05) and positive correlation between iron concentration and age (*r* = 0.28, *p* < 0.01). HbA1c was positively associated with MCV (*r* = 0.31, *p* < 0.01) and negatively with MCHC (*r* = −0.33, *p* < 0.001). Additionally, statistical analysis revealed some significant correlations of the analyzed biochemical and blood count parameters with BMI SDS in T1D patients. Vitamin B12 concentration (*r* = −0.27, *p* < 0.01) (Figure 1) and MCHC (*r* = −0.23, *p* < 0.05) were inversely related to BMI SDS. Furthermore, a positive correlation was determined with TIBC (*r* = 0.28, *p* < 0.01) (Figure 2) and HbA1c (*r* = 0.37, *p* < 0.001).

Neither T1D duration nor the percent of life with T1D was found to be related to BMI SDS.

## 4. Discussion

Until present, there have not been many studies assessing the prevalence of anemia among children with T1D. There is a range of publications regarding patients with type 2 diabetes and anemia, usually accompanying renal complications [11, 12].

None of the investigated children in this study had anemia. Thomas et al. estimated that the prevalence of anemia (based on the hemoglobin concentration) in a group of adults having T1D for a mean of 20 years is equaled to 14%. Risk factors included disturbed renal function and albuminuria (anemia was present in 52% of patients with macroalbuminuria, 24% with microalbuminuria, and only <8% of individuals with normal albumin secretion) [13]. The mentioned study included adults with long lasting diabetes, in which renal complications had already developed. In the pediatric population, disturbances of renal function are rare. Children remain under strict parental and specialist medical care. Regular control visits and glycemic control (HbA1 measurements) as well as education allow achieving optimal diabetes control and avoiding renal complications. Children enrolled for this study according to their medical records did not have diagnosis of micro- or macroalbuminuria. Although urine albumin secretion was not measured at the day when blood for laboratory test was drawn, all patients are followed in the outpatient clinic according to the recommendations of Diabetes Poland and have serum creatinine and urine albumin measured every 1-2 years and blood pressure at every control visit.

In an analysis of 200 children with T1D conducted in Egypt, anemia was diagnosed in 37% of cases. Among patients with anemia, 54.7% had iron deficiency, 18.7% had folic acid deficiency, 18.7% were diagnosed with thalassemia minor, 4% with celiac disease, and in 24% anemia developed as a consequence of a parasitic infestation [14]. Mean age of the analyzed patients (11.2 years) and the duration of T1D (mean 4 years) are similar to that of our patients. However, such high prevalence of anemia in Egyptian children may result from a different socioeconomic situation of this region leading to cases of nutritional deficits (folic acid and/or iron deficiency) and hygienic negligence (parasitic infestations). Such frequent cases of thalassemia minor is related to its general higher prevalence in the region of the Mediterranean Sea.

In another investigation, coming from Poland, patients with newly diagnosed T1D revealed a tendency for iron deficiency anemia in comparison to children with longer lasting T1D (>1 year) [15].

The mean values of iron concentration, iron metabolism parameters, and vitamin B12 levels were within normal ranges in our patients. Normal vitamin B12 and iron concentrations in children with T1D were also described by other authors [14, 16].

Adequate blood count results, iron concentrations, iron metabolism parameters, and vitamin B12 levels in our patients can be explained by optimal nutrition and regular control visits at the outpatient clinic as well as continuous reeducation. Despite results remaining within normal ranges, we revealed statistically significant differences in terms of RDW, TIBC, and hepcidin in comparison to the control subjects, which partially confirms the initial research hypothesis. RDW is a sensitive indicator of a developing anemia. Its normal value reflects isocytosis. Increased RDW (anisocytosis) occurs not only in cases of iron deficiency but also in anemia related to inflammation, although the values are higher in iron deficiency anemia [17]. Hepcidin is a systemic regulator of iron homeostasis. It is produced in the liver as a result of iron stimulation as well as in inflammatory states (IL-6 stimulation). Its role is to stop iron in the intracellular poll and decrease iron absorption from the gastrointestinal tract. As a result serum iron concentration decreases. In inflammatory states caused by infection, this is a beneficial defensive mechanism, which hides iron from being used by bacteria. Nevertheless, in chronic inflammation, it may lead to inflammation-related anemia [5]. Higher RDW in comparison to the control group, higher hepcidin concentration, and lower TIBC may suggest a trend towards typical changes of observed anemia subsequent to chronic inflammation. It could be hypothesized/speculated that in these children there was an occult, asymptomatic, and chronic inflammation or such changes in the measured parameters are a consequence of other unidentified factors. This requires further investigation and possibly also a follow-up to see whether this trend of parameter results is sustained.

Because of the increasing global problem concerning improper body weight in children, also those with T1D [18], as well as higher prevalence of overweight and obesity among young adults with T1D (aged 20–40 years old) in comparison to healthy individuals [19], we analyzed the correlations between BMI SDS and the assessed red blood cell and biochemical parameters. Frequency of overweight in children with T1D in past publications was approximated as high as 30% [20, 21]. In our patients with T1D, BMI SDS > 1 SDS was revealed in 21% of cases. The publications concerning the relationship between overweight and anemia in children with T1D, according to our knowledge, are lacking. However, studies on the obese population are available. In our study BMI SDS values correlated negatively with vitamin B12 concentrations. This is unequivocal with the results of analyses of 1252 patients of an obesity clinic, who were diagnosed with severe obesity (BMI > 40 kg/m^2^) [22]. The authors revealed also a negative association of BMI and vitamin B12 levels. In the mentioned study, vitamin B12 deficiency was revealed in as many as 20.9%. It is interesting that iron deficiency in this group of patients was present in only 9.8% subjects [22]. Taking into consideration the above information in obese patients, in the first line, not iron deficiency but rather vitamin B12 deficiency should be suspected. This is probably related to improper dietary habits of the obese patients—ingestion of foods with high carbohydrate and fat content and restriction of consumption of valuable products. In our study, iron and vitamin B12 concentrations were within normal ranges. This fact may result from a much smaller number of obese individuals in our study group. Additionally, none of our patients had severe obesity. T1D children are under parental care, and a balanced diet is crucial part of diabetes therapy. Nevertheless, the inverse association could be confirmed. We did not reveal a direct relation between BMI SDS and iron concentration, which is similar to the results of other publications [22]. The results revealed, however, a positive association of BMI SDS and TIBC, a parameter often used in iron metabolism disorders, and an inverse relation with MCHC. These associations are not surprising, as the results of a meta-analysis confirmed a strong relation of obesity and iron deficiency [23].

It is also a known fact that T1D patients with improper body weight often do not have optimal glycemic control [24, 25]. In our study, it is reflected by a positive correlation of BMI SDS and HbA1c.

Expanded diagnostic tests conducted in a representative group of children and adolescents with T1D allowed to construct a reliable database. Another strength of this study is the enrollment of patients without other concomitant autoimmune diseases which could interfere the final results as well as the comparison with a healthy control group. The analysis of the iron metabolism parameters was precise and complex, including the assessment of hepcidin, which is not often measured in T1D children. As a limitation, the quite wide range of diabetes duration (recently diagnosed to long lasting) should be mentioned.

## 5. Conclusions

The assessed blood count, iron metabolism parameters, and vitamin B12 results remained in the studied patients within normal ranges. Therefore, single assays of the abovementioned basic parameters may not be sufficient indicators of anemia in children with T1D.

However, results remaining within reference ranges do not exclude a patient with T1D from being at risk of the development of anemia. Basing on statistically significant differences among respective parameters of the blood count and iron metabolism in children with T1D as compared with the control group, it is indicated to monitor trends, and the interpretation of results should be holistic and simultaneous.

In the schedule of the health care of children with T1D, in addition to monitoring development of celiac disease and thyroid dysfunction, it should also be considered to follow alteration in the blood count, iron metabolism, and vitamin B12 status.

Moreover, in children with T1D and abnormal body weight, coexisting deficiencies of vitamin B12 and iron should be taken into account.

## Figures and Tables

**Table 1:** Age, clinical parameters related to type 1 diabetes (T1D), complete blood count and serum parameters results in all patients with T1D and in the control group. Parameters are expressed as mean value and its 95% confidence interval.

		T1D (*N* = 94)	Controls (*N* = 43)
Clinical parameters	Age (yrs)	12.9 (12.1, 13.8)	13.1 (12.0, 14.3)
	Age at T1D diagnosis (yrs)	8.7 (7.9, 9.5)	
	T1D duration (yrs)	4.2 (3.5, 4.9)	
	HbA1c (%)	7.34 (7.02, 7.65)	

Complete blood count	RBC (10^6^/*μ*l)	4.72 (4.64, 4.79)	4.76 (4.64, 4.89)
	MCV (fl)	86.5 (85.7, 87.3)	85.5 (84.2, 86.8)
	MCHC (g/dl)	33.8 (33.7, 34.0)	34.0 (33.7, 34.3)
	Hemoglobin (g/dl)	13.79 (13.58, 13.99)	13.83 (13.46, 14.21)
	Hematocrit (%)	40.8 (40.0, 41.4)	40.7 (39.7, 41.7)
	RDW (%)	13.6 (13.3, 13.8)^∗^	12.6 (12.4, 12.9)^∗^

Serum parameters	Iron (*μ*mol/l)	17.34 (16.19, 18.49)	16.40 (14.19, 18.60)
	TIBC (*μ*mol/l)	59.09 (57.51, 60.67)^∗^	68.15 (65.29, 71.02)^∗^
	Ferritin (*μ*g/l)	48.51 (41.93, 55.08)	45.87 (35.21, 56.53)
	Transferrin (g/l)	3.01 (2.47, 3.56)	3.01 (2.83, 3.19)
	Vitamin B12 (pg/ml)	459 (419, 500)^∗^	397 (356, 437)^∗^
	Hepcidin (ng/ml)	0.25 (0.20, 0.29)^∗^	0.12 (0.10, 0.14)^∗^

T1D: type 1 diabetes, HbA1c: glycated hemoglobin A1c, RBC: red blood cells, MCV: mean corpuscular volume, MCHC: mean corpuscular hemoglobin concentration, RDW: red blood cell distribution width, TIBC: total iron-binding capacity. ^∗^Significant difference between T1D and controls (RDW *p* < 0.001; vitamin B12 *p* < 0.01; hepcidin *p* < 0.001; TIBC *p* < 0.001).

**Table 2:** Logistic regression—final model (controls versus T1D). Columns 2–4 present coefficient estimates, their standard error, and *p* value; columns 5-6 adjusted odds ratio (OR) and its 95% confidence interval (CI).

Final model components	*β* estimate	Standard error	*p* value	OR	95% CI
Intercept	−4.42	4.85	0.3621		
RDW	1.08	0.37	0.0034	2.94	(1.43; 6.06)
Hepcidin	11.24	4.41	0.0108	7.6*e* + 04	(13.38; 4.3*e* + 08)
TIBC	−0.17	0.04	3.4*e* − 06	0.85	(0.79; 0.91)

**(a):** 

Index	True positive TP	True negative TN	False negative FN	False positive FP	Total
Classification results	84	33	10	10	137

**(b):** 

Index	Sensitivity	Specificity	Accuracy
Classification results	89.4%	76.7%	85.4%

## Data Availability

The data generated or analyzed during this study are included in this published article. Requests for material should be made to the corresponding author.

## References

[1] Kahaly G. J., Hansen M. P. (2016). Type 1 diabetes associated autoimmunity. *Autoimmunity Reviews*.

[2] Patterson C. C., Dahlquist G. G., Gyürüs E., Green A., Soltész G. (2009). Incidence trends for childhood type 1 diabetes in Europe during 1989–2003 and predicted new cases 2005–20: a multicentre prospective registration study. *The Lancet*.

[3] Angelousi A., Larger E. (2015). Anaemia, a common but often unrecognized risk in diabetic patients: a review. *Diabetes & Metabolism*.

[4] Soliman A. T., De Sanctis V., Yassin M., Soliman N. (2017). Iron deficiency anemia and glucose metabolism. *Acta Bio Medica Atenei Parmensis*.

[5] Jackowska T., Wójtowicz J. (2014). Niedokrwistość chorób przewlekłych. *Postępy Nauk Medycznych*.

[6] Cotroneo P., Maria Ricerca B., Todaro L. (2000). Blunted erythropoietin response to anemia in patients with type 1 diabetes. *Diabetes/Metabolism Research and Reviews*.

[7] Christy A. L., Manjrekar P. A., Babu R. P., Hegde A., Rukmini M. S. (2014). Influence of iron deficiency anemia on hemoglobin A1c levels in diabetic individuals with controlled plasma glucose levels. *Iranian Biomedical Journal*.

[8] Craig M. E., Jefferies C., Dabelea D., Balde N., Seth A., Donaghue K. C. (2014). ISPAD clinical practice consensus guidelines 2014. Definition, epidemiology, and classification of diabetes in children and adolescents. *Pediatric Diabetes*.

[9] Clinical Diabetology (2018). 2018 guidelines on the management of diabetic patients. A position of Diabetes Poland. *Clinical Diabetology*.

[10] Jeffreys H. (1961). *The Theory of Probability*.

[11] Ito H., Takeuchi Y., Ishida H. (2010). Mild anemia is frequent and associated with micro- and macroangiopathies in patients with type 2 diabetes mellitus. *Journal of Diabetes Investigation*.

[12] Ezenwaka C. E., Jones-LeCointe A., Nwagbara E., Seales D., Okali F. (2008). Anaemia and kidney dysfunction in Caribbean type 2 diabetic patients. *Cardiovascular Diabetology*.

[13] Thomas M. C., MacIsaac R. J., Tsalamandris C. (2004). Anemia in patients with type 1 diabetes. *The Journal of Clinical Endocrinology & Metabolism*.

[14] Salah N., el Hamid F. A., Abdelghaffar S., el Sayem M. (2005). Prevalence and type of anaemia in young Egyptian patients with type 1 diabetes mellitus. *Eastern Mediterranean Health Journal*.

[15] Wójciak R. W., Mojs E., Stanisławska-Kubiak M. (2014). The occurrence of iron-deficiency anemia in children with type 1 diabetes. *Journal of Investigative Medicine*.

[16] Forte G., Bocca B., Peruzzu A. (2013). Blood metals concentration in type 1 and type 2 diabetics. *Biological Trace Element Research*.

[17] Johnson M. A. (1990). Iron: nutrition monitoring and nutrition status assessment. *The Journal of Nutrition*.

[18] Frohlich-Reiterer E. E., Rosenbauer J., Bechtold-Dalla Pozza S. (2014). Predictors of increasing BMI during the course of diabetes in children and adolescents with type 1 diabetes: data from the German/Austrian DPV multicentre survey. *Archives of Disease in Childhood*.

[19] Szadkowska A., Madej A., Ziółkowska K. (2015). Gender and age-dependent effect of type 1 diabetes on obesity and altered body composition in young adults. *Annals of Agricultural and Environmental Medicine*.

[20] Łuczyński W., Szypowska A., Głowińska-Olszewska B., Bossowski A. (2011). Overweight, obesity and features of metabolic syndrome in children with diabetes treated with insulin pump therapy. *European Journal of Pediatrics*.

[21] Luczyński W., Szypowska A., Bossowski A. (2010). Overweight, obesity and metabolic syndrome in children with type 1 diabetes mellitus. *Pediatric Endocrinology, Diabetes, and Metabolism*.

[22] Arshad M., Jaberian S., Pazouki A., Riazi S., Rangraz M. A., Mokhber S. (2017). Iron deficiency anemia and megaloblastic anemia in obese patients. *Romanian Journal of Internal Medicine*.

[23] Zhao L., Zhang X., Shen Y., Fang X., Wang Y., Wang F. (2015). Obesity and iron deficiency: a quantitative meta-analysis. *Obesity Reviews*.

[24] Jose L. P., Cardoso-Demartini Ade A., Liberatore Junior R. D. (2009). Clinical and laboratory profile of pediatric and adolescent patients with type 1 diabetes. *Jornal de Pediatria*.

[25] Wysocka-Mincewicz M., Kołodziejczyk H., Wierzbicka E., Szalecki M. (2015). Overweight, obesity and lipids abnormalities in adolescents with type 1 diabetes. *Pediatric Endocrinology Diabetes and Metabolism*.

